# A Proxy for Detecting IUGR Based on Gestational Age Estimation in a Guatemalan Rural Population

**DOI:** 10.3389/frai.2020.00056

**Published:** 2020-08-07

**Authors:** Camilo E. Valderrama, Faezeh Marzbanrad, Rachel Hall-Clifford, Peter Rohloff, Gari D. Clifford

**Affiliations:** ^1^Department of Biomedical Informatics, Emory University, Atlanta, GA, United States; ^2^Department of Electrical and Computer Systems Engineering, Monash University, Melbourne, VIC, Australia; ^3^Department of Sociology, Center for the Study of Human Health, Emory University, Atlanta, GA, United States; ^4^Wuqu' Kawoq | Maya Health Alliance, Santiago Sacatepéquez, Guatemala; ^5^Division of Global Health Equity, Brigham and Women's Hospital, Boston, MA, United States; ^6^Department of Biomedical Engineering, Georgia Institute of Technology, Emory University, Atlanta, GA, United States

**Keywords:** gestational age estimation, one-dimension Doppler ultrasound (1D-DUS), fetal heart rate (FHR), intra-uterine growth restriction (IUGR), maternal blood pressure, signal processing, supervised machine learning, low-and middle-income countries (LMICs)

## Abstract

*In-utero* progress of fetal development is normally assessed through manual measurements taken from ultrasound images, requiring relatively expensive equipment and well-trained personnel. Such monitoring is therefore unavailable in low- and middle-income countries (LMICs), where most of the perinatal mortality and morbidity exists. The work presented here attempts to identify a proxy for IUGR, which is a significant contributor to perinatal death in LMICs, by determining gestational age (GA) from data derived from simple-to-use, low-cost one-dimensional Doppler ultrasound (1D-DUS) and blood pressure devices. A total of 114 paired 1D-DUS recordings and maternal blood pressure recordings were selected, based on previously described signal quality measures. The average length of 1D-DUS recording was 10.43 ± 1.41 min. The min/median/max systolic and diastolic maternal blood pressures were 79/102/121 and 50.5/63.5/78.5 mmHg, respectively. GA was estimated using features derived from the 1D-DUS and maternal blood pressure using a support vector regression (SVR) approach and GA based on the last menstrual period as a reference target. A total of 50 trials of 5-fold cross-validation were performed for feature selection. The final SVR model was retrained on the training data and then tested on a held-out set comprising 28 normal weight and 25 low birth weight (LBW) newborns. The mean absolute GA error with respect to the last menstrual period was found to be 0.72 and 1.01 months for the normal and LBW newborns, respectively. The mean error in the GA estimate was shown to be negatively correlated with the birth weight. Thus, if the estimated GA is lower than the (remembered) GA calculated from last menstruation, then this could be interpreted as a potential sign of IUGR associated with LBW, and referral and intervention may be necessary. The assessment system may, therefore, have an immediate impact if coupled with suitable intervention, such as nutritional supplementation. However, a prospective clinical trial is required to show the efficacy of such a metric in the detection of IUGR and the impact of the intervention.

## 1. Introduction

Estimation of fetal gestational age (GA) provides important information throughout pregnancy, such as delivery scheduling, growth disorder detection, and preterm newborns management (Alexander et al., 1995). Thus, GA estimation can assist in detecting issues leading to perinatal mortality and morbidity (Rijken et al., 2014; Karl et al., 2015). This detection is particularly needed in low-and middle-income countries (LMICs), which account for ~98% of all reported perinatal deaths worldwide, largely due to gestational developmental issues (Zupan, 2005).

In high-income countries, clinical teams generally use ultrasound images to estimate GA, as well as any structural abnormalities (Malhotra et al., 2014). These GA estimations are based on a variety of fetal measurements, such as biparietal diameter, crown-rump length, head circumference, abdominal circumference, and femur length (Malhotra et al., 2014). However, in LMICs, the access to ultrasound imaging is limited, and almost unavailable in rural areas, due to the high cost of the medical equipment, the expenses for maintenance, and the requirement of skilled medical staff (World Health Organization, 2014). Hence, low-cost alternative methods for dating gestation are used in LMICs.

A common low-cost method used for GA estimation is the last menstrual period (LMP), in which a 28-days menstrual cycle is assumed. Although previous studies have criticized LMP due to the inconsistency in the menstrual cycle length (Dietz et al., 2007), and the difficulty to recall the day of the last menstrual period (Andersen et al., 1981), the LMP method has shown to be a somewhat useful method for LMICs, particularly in rural areas lacking medical equipment. In fact, Neufeld et al. (2006) compared 171 GA estimations based on LMP collected in rural Guatemala with GA estimations given by biparietal diameter, reporting that GA estimations by the LMP were within ±14 days of the biparietal diameter estimations for 94% of the cases.

GA estimations based on LMP can assist in the assessment of intrauterine growth restriction (IUGR), which has a prevalence varying between 9 and 11% in LMICs (de Onis et al., 1998; Lee et al., 2013). Specifically, IUGR is assessed by comparing the estimate of GA with the symphysis-fundal height measurement (World Health Organization, 2016). For fetuses growing normally, from 24 weeks of gestation, the symphysis-fundal height measurement (*L*_*sfh*_) in centimeters should correspond to the number of weeks of gestation ±2 cm. When *L*_*sfh*_ < *N* − 2, where *N* is the number of weeks since the last menstrual period, the fetus is suspected to be IUGR (Peter et al., 2015). However, the symphysis-fundal height method lacks significant evidence to recommend its widely use in LMICs (World Health Organization, 2016). Moreover, previous studies have noted that the SHF has exhibited a large error of ±6 weeks for estimating GA (Griffiths et al., 2008). New approaches are, therefore, still needed to provide reproducible and low-cost assessment for detecting abnormal growth in settings in which ultrasound images, taken by trained operators, are not available.

In this work, we propose an alternative approach for GA estimation to provide a proxy for assessing fetal development and identifying possible cases of IUGR for a Guatemalan rural population, in which ultrasound imaging is not affordable and the symphysis-fundal height is not accurate. Our approach estimates GA using fetal heart rate variability (fHRV) indexes and maternal hemodynamics derived from one-dimensional Doppler ultrasound (1D-DUS) and maternal blood pressure, respectively. Data were acquired during routine perinatal check-up visits by traditional birth attendants using a low-cost Doppler transducer and a self-inflating blood pressure device (Stroux et al., 2016; Martinez et al., 2017, 2018). These features were used to build a machine learning algorithm to estimate GA. We hypothesized that if the estimated GA is lower than the GA calculated from last menstruation, then this could be interpreted as a potential sign of IUGR based on low birth weight (LBW), and referral and intervention may be necessary.

## 2. Background

**Figure 1:**
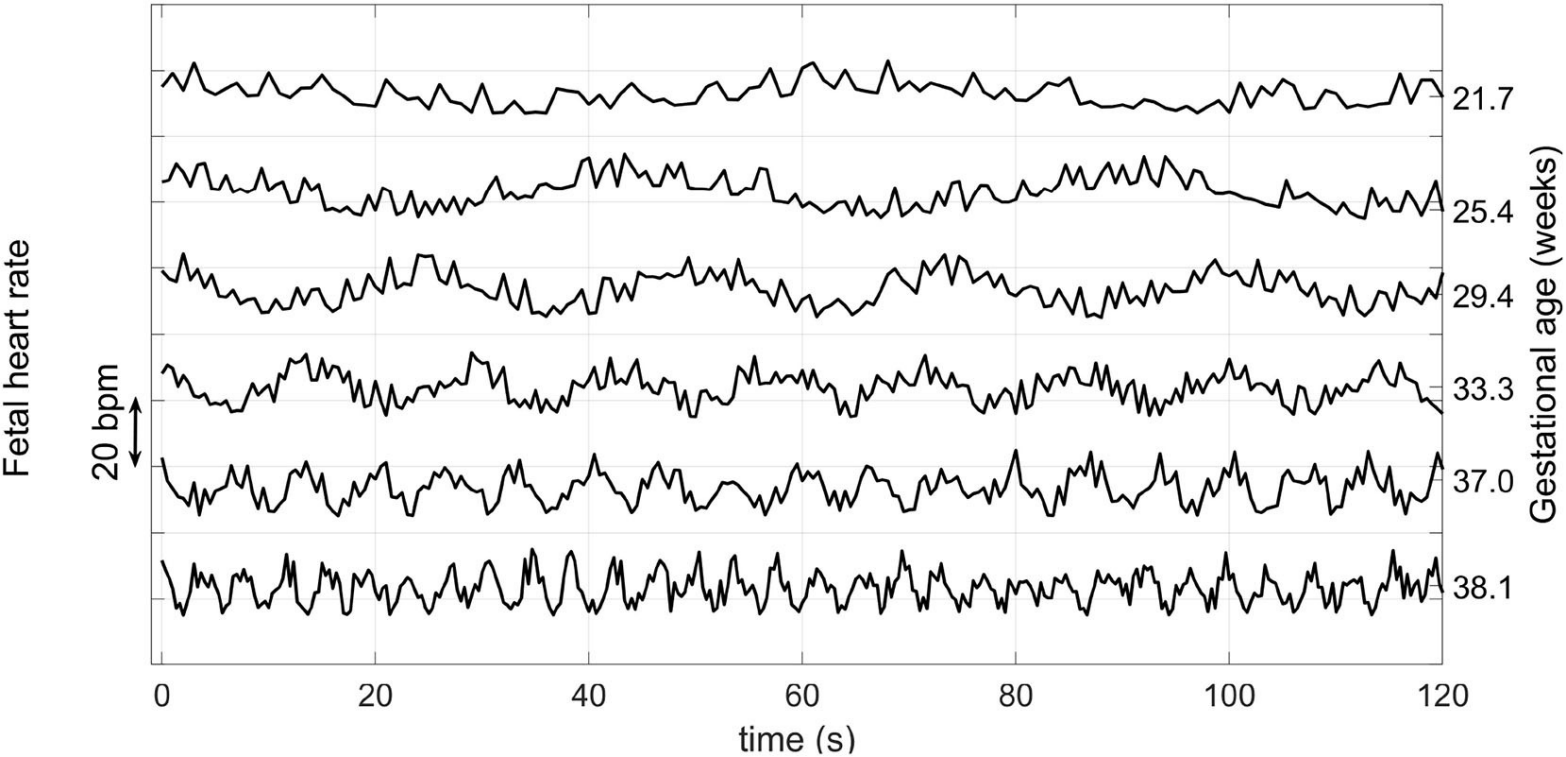
Variation of the FHR from 22 to 38 weeks during pregnancy. Note that the vertical axis has an arbitrary offset. Average FHR does not drop by such a large amount each week during pregnancy, but rather it drops on average by about 15 BPM from week 25 to 40 (Kapaya et al., 2018). Adapted from Wakai (2004).

Fetal heart rate is influenced by the Autonomic Nervous System (ANS) (Schneider et al., 2009; Wallwitz et al., 2012), which in turn modifies FHR dynamics over the course of pregnancy. In particular, FHR variability evolves over the course of pregnancy and may reflect the maturity of the ANS, and thus may indicate the fetal GA. Wakai (2004) reported that fHRV, as observed from traces taken from 61 pregnant women without complications, increases during gestation. In particular, they noted that short term variability increased during the last trimester, whereas long term variability exhibited the largest increases in the early gestational period. Figure 1 shows an example of how FHR changes across gestation, as reported in Wakai (2004).

Based on FHR, previous studies have shown a correlation between GA and markers derived from fHRV. Linear metrics, such as the mean of R-R interval, the standard deviation and root mean square of successive differences positively correlated with GA for both genders (Lange et al., 2005). Non-linear metrics, such as approximate entropy (ApEn), Lyapunov exponent, tone-entropy and generalized mutual information, have also been linked to fetal maturation (Van Leeuwen et al., 1999; Hoyer et al., 2012; Khandoker et al., 2015). Additionally, Van Leeuwen et al. (2003) and Signorini et al. (2003) reported that power in the 0.003–1.0 Hz frequency band vary during pregnancy.

Initial works on GA estimation aimed to find a relation between GA and FHR-based metrics using univariate regression (Hoyer et al., 2012). More recently, some works have aimed to improve the characterizing of FHR by incorporating multivariate and more complex methods. In particular, Tetschke et al. (2016) extracted features from 359 high resolution fetal magnetocardiographic recordings, lasting at least 20 min. The researchers implemented an algorithm to extract non-active portions of the recording and calculated both linear and non-linear metrics of fHRV from the these *quiet* periods. Results showed that entropy and skewness were more highly correlated with GA than those obtained by traditional linear HRV metrics. However, this approach requires high temporal and spatial resolution data acquired from costly and non-portable equipment, making its use in LMICs impractical.

In earlier work, Marzbanrad et al. (2016) estimated GA for 57 fetuses using a step-wise regression based on cardiac wall intervals derived from one-dimension Doppler ultrasound signal (1D-DUS) and fECG signals recorded in a Japanese hospital. The estimated GAs were compared to the GA derived from crown-rump length, achieving a mean square error of 3.8 and 5.1 weeks for cardiac intervals and fHRV parameters, respectively. In further work, Marzbanrad et al. (2017) improved the estimation accuracy by incorporating 1D-DUS and fECG quality assessment algorithms to filter poor quality signals. As a result, the step-wise regression achieved a mean absolute error (MAE) of 4.7 weeks from fHRV parameters, and 2.7 weeks when including the cardiac intervals metrics. Although this latter method achieved comparable results to Doppler imaging based estimations, it required two sources, 1D-DUS and fECG signals, which increases costs and complicates implementation, particularly in LMICs (Stroux et al., 2016).

In addition to FHR indexes, maternal blood pressure is also a relevant metric for GA estimation. Previous works have reported that maternal systolic and diastolic blood pressure increases throughout pregnancy (Steer et al., 2004; Salas et al., 2006; Kac et al., 2015; Rebelo et al., 2015). However, despite the correlation between GA and maternal blood pressure, no research has included maternal blood pressure in regression models to estimate GA. We note that extreme blood pressures may be indicative of pre-eclampsia, or other gestational issues. It is therefore important to treat these separately.

## 3. Methods

### 3.1. Databases

#### 3.1.1. Collection of the Data

Data used in this work were collected as a part of a randomized control trial conducted in rural highland Guatemala in the vicinity of Tecpan, Chimaltenango. This program was approved by the Institutional Review Boards of Emory University, the Wuqu' Kawoq | Maya Health Alliance, and Agnes Scott College (Ref: IRB00076231—“Mobile Health Intervention to Improve Perinatal Continuum of Care in Guatemala”) and registered on ClinicalTrials.gov (identifier NCT02348840). In the trial, traditional birth attendants were trained to use a mobile mHealth system to record perinatal information during approximately monthly visits during the second and third trimesters. More details on the design and implementation of the mobile mHealth system, and the training of the traditional birth attendants can be found in Stroux et al. (2016) and Martinez et al. (2017, 2018). At this time, the dataset is not publicly available; however, a de-identified dataset can be available upon request and approbation of the project ethical committee.

The perinatal care program included both prenatal and post-partum visits. In the prenatal visits, traditional birth attendants recorded GA in months by counting the number of whole months since the last menstrual period. The GA was recorded in months instead of weeks to reduce measurement errors since usually patients attended in this project forgot the specific date of their last menstrual period, and very few received an early obstetrical ultrasound for more accurate dating (Martinez et al., 2017). During the visit, the traditional birth attendants also recorded 1D-DUS signals and blood pressure from the mother in a supine position using the mobile mHealth system (Stroux et al., 2016; Martinez et al., 2017, 2018). The 1D-DUS signals were recorded using a Doppler transducer (AngelSounds Fetal Doppler JPD-100s, Jumper Medical Co., Ltd., Shenzhen, China) with an ultrasound transmission frequency of 3.3 MHz and a digitization sampling frequency of 44.1 kHz. The maternal blood pressure was taken from both arms using a self-inflating blood pressure device calibrated for pregnancy.

In the post-partum visits, traditional birth attendants recorded the newborn's birth date, sex, current weight, length, and head size. These post-partum visits could occur days or months after birth since sometimes it was difficult to follow up on the patients. The birth weight was then estimated using a Reed2 second-order model (Berkey and Reed, 1987) fitted on 917 observed post-natal weights using an approach we have previously described in Valderrama et al. (2020). A weight threshold was used to classify the estimated birth weights as low or normal. This threshold was defined by first finding the percentile corresponding to ≤2.5 kg in a Guatemalan national maternal survey for the region of relevance in our study (MSPAS/Guatemala et al., 2017). We found that the lowest 14.3% of male newborns and 16.33% of female newborns satisfied this weight criterion. These percentiles were then located in our estimated birth weight distribution to determine the corresponding LBW threshold. This corresponded to 2.64 kg for males and 2.57 kg for females.

#### 3.1.2. Assumption of the Study

In this work, a newborn was considered as a possible case of IUGR if their estimated birth weight was below the threshold discussed above. This assumption is based on the fact that LBW could be a consequence of either preterm birth (<37 weeks) or small-for-gestational-age. However, in LMICs, around 60% of LBW newborns are small-for-gestational-age (Lee et al., 2013), and the main reason for small-for-gestational-age in LMICs is IUGR (de Onis et al., 1998; Lee et al., 2013).

#### 3.1.3. Data Inclusion Criteria

Prenatal visits were included if they contained both blood pressure pictures and 1D-DUS recordings with some conditions. For the blood pressure, the numbers had to be readable on the photograph of the blood pressure device. Also, the difference between the right and left arm measurements had to be lower than 15 mmHg, thus discarding any spurious measurements. Finally, possible preeclampsia patients were discarded when systolic or diastolic blood pressure was higher than a threshold. This threshold was defined at 130 and 80 mmHg for SBP and DBP, as is suggested for measurements taken in spine position (Netea et al., 2003; Kluttig et al., 2010; Cicolini et al., 2011).

The conditions for including the 1D-DUS recording were based on length and quality. The minimum length was fixed at 10 min since earlier work suggests that this is the required length to extract fHRV indexes, such as baseline, accelerations, and decelerations (Dobbe et al., 2001).

In addition to the length, the quality of a 1D-DUS recording was also considered as an inclusion criterion. The 1D-DUS quality was assessed using a window of 3.75 s and a sliding window of 250 ms. For each 3.75-s window, 16 features were extracted, including Wavelet percentage energy in the range 250–2,000 Hz, Mel-frequency cepstral coefficients, and power spectrum ratios on electrical interference frequency ranges. The features were fed into a classifier composed of a logistic regression and a multiclass support vector machine to classify the 3.75-s window into good quality, interference, silence, talking in the background, or low signal to noise ratio. More details of the quality assessment method can be found in Valderrama et al. (2017, 2018a).

Based on the length and quality criteria, a 1D-DUS recording was only included in this present work if it lasted more than 10 min, and at least 50% of its 3.75-s windows were labeled as good quality.

#### 3.1.4. Final Data Set

After applying the inclusion criteria, the final dataset comprised 167 visits from 153 non-preeclampsia women who were pregnant with singleton fetuses. From these patients, 142 gave birth to normal weight singletons, whereas 24 gave birth to LBW newborns, based on our thresholds defined for the study population (Valderrama et al., 2020) (see subsection 3.1.1).

Table 1 shows demographics of the patients. The male/female ratio was higher in the LBW group than in the normal birth weight group. On the other hand, the maternal age and the number of previous pregnancies (gravidity) were higher in the normal weight group.

**Table 1:** Average demographics for the data used in this study.

**Variable**	**Normal birth weight**	**Low birth weight**
Patients (count)	129	24
Newborn gender (male/female)	56/73	17/7
Birth weight (kg)	3.1 (*SD* = 0.3; *N* = 129)	2.3 (*SD* = 0.4; *N* = 24)
Maternal age (years)	27.0 (*SD* = 6.3; *N* = 123)	24.5 (*SD* = 6.7; *N* = 22)
Gravidity (count)	3.4 (*SD* = 2.5; *N* = 96)	2.3 (*SD* = 3.1; *N* = 16)

*For each metric, the standard deviation and the number of patients available for that variable are shown in parenthesis*.

Table 2 shows the distribution of the GA based on the last menstrual period (LMP) method. Visits ranged from the sixth to the ninth month of pregnancy, focusing mainly on the third trimester.

**Table 2:** Number of visits per gestational age (GA) taken with the last menstrual period (LMP) method.

**Gestational age (months)**	**Normal weight**	**Low birth weight**
6	9	3
7	38	7
8	37	5
9	58	10
Total	142	25

### 3.2. Approach Overview

**Figure 2:**
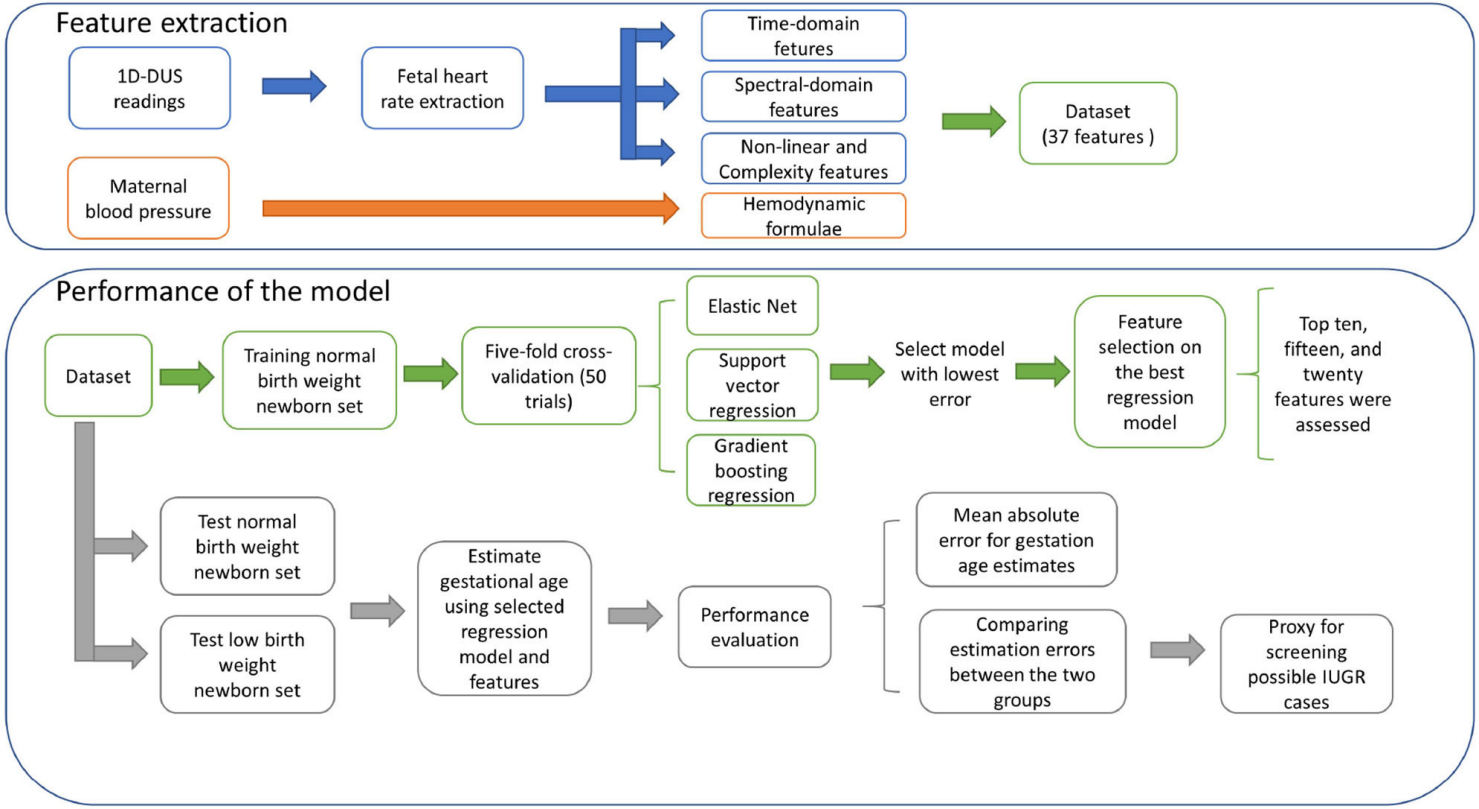
Block diagram of the approach for detecting possible cases of IUGR.

Figure 2 shows an overview of the approach used to detect possible cases of IUGR. First, 37 features were extracted from the raw 1D-DUS signals and maternal blood pressure readings. The extracted dataset was split into three subsets: normal birth weight (NBW) training set, NBW test set, and LBW test set. The training set was used to select the best regression model and features. The selected regression model and features were used to estimate GA from the NBW and LBW held-out test sets. The GA estimate errors of the NBW and LBW were compared to assess if birth weight affected the GA estimation. The following subsections provide details of each component of the approach.

### 3.3. Deriving the FHR Signal

#### 3.3.1. Extracting Fetal Heart Rate From 1D-DUS

Each 1D-DUS recording was analyzed using a window of 3.75 s and a sliding window of 0.25 s. The window length was set at 3.75 s since it is the usual length for computerized analysis of fetal non-stress tests based on the Dawes/Redman criteria (Dawes et al., 1981; Pardey et al., 2002). The selection of the sliding window was based on the desired sampling frequency, namely 4 Hz. This sampling frequency has been shown to be sufficient for digital cardiotocography (Romagnoli et al., 2019), and corresponded to a Nyquist frequency of 2 Hz, thus allowing the extraction of spectral metrics in the range 0.03–1 Hz.

For each 3.75-s window, the fetal heart rate (FHR) was estimated auto-correlation (AC)-based method using an open source code written in Matlab (MathWorks, Natick, MA, USA), previously introduced in (Valderrama et al., 2018b, 2019). Specifically, the method detects the fundamental period of the envelope of the 3.75-s window by applying auto-correlation, and then the FHR is estimated by dividing 60 between the fundamental period in seconds. More details of the FHR estimator are found in (Valderrama et al., 2019).

In addition to estimate the FHR, the quality of the 3.75-s window was also assessed and stored for further prepossessing steps. The quality was assessed using the method presented in Valderrama et al. (2018a) (see subsection 3.1.3).

#### 3.3.2. Pre-processing of Estimated FHR Signal

Since 1D-DUS recordings are prone to noise, any given 3.75-s window of Doppler data may lead to an unreliable estimate. Two steps then assessed the reliability of the extracted FHR. Firstly, as recommended in Nyboe (2011), we removed FHR estimates that were not within the 65–175% range of the average of the previous two estimates. Secondly, we removed 3.75-s windows classified as something else other than good quality.

Each discarded value was replaced by the linear interpolation between its previous point and the next stable segment. A stable segment was defined as a region of five adjacent points for which the FHR estimate did not vary by more than ten beat per minute (BPM).

#### 3.3.3. Calculation of the Baseline, Acceleration, and Deceleration

The baseline was determined using an algorithm proposed by Andersson (2011), which is an improvement of those proposed by Dawes et al. (1982) and Mantel et al. (1990). Specifically, a filter bank was applied to the 4-Hz FHR time series to attenuate any accelerations or deceleration.

Following the work of Andersson (2011), accelerations and decelerations were detected for each valid 1-min segment of the baseline. A valid segment was determined by computing a FHR histogram using a bin width of 10 BPM. If the most frequent bin of the histogram contained more than 40% of the values, the 1-min baseline segment was considered valid.

When a 1-min baseline segment was determined to be valid, acceleration and deceleration intervals were identified following Dawes criteria (Dawes et al., 1982). Namely, an acceleration was defined to be a section of data for which the FHR was higher than the baseline for at least 15 *s* and at least one sample was 15 BPM or more above the baseline. Similarly, a deceleration was defined where the FHR remained below the baseline FHR for at least 15 s, and at least one sample was 15 BPM below the baseline. More details of the algorithm can be found in Andersson (2011).

### 3.4. Features Used for Gestational Age Estimation

Based on previous works presented in section 2, a total of 37 features relevant for estimating GA were extracted from the 1D-DUS and blood pressure device captured at the perinatal visits.

#### 3.4.1. Features Extracted From the 1D-DUS

The features derived from 1D-DUS recording were calculated using the FHR time series (see section 3.3). Since the RR-interval sequence is necessary to estimate fHRV metrics, the FHR series was converted into a interbeat sequence as:

(1) $$\begin{array}{r} T\left(i\right)=60,000/S\left(i\right), \end{array}$$

where *S*(*i*) is the FHR at the *i* − *th* second.

Table 3 shows the total features extracted from the FHR time series and the interbeat sequence. The table also provides the physiological interpretation for each feature and relevant previous works in which such features have been used to assess gestational development. The features were grouped into three categories: linear time-domain, non-linear and complexity, and frequency-domain features.

**Table 3:** Features extracted from 1D-DUS signals.

**Feature**	**Calculation**	**Interpretation**	**Relevant previous works**
**LINEAR TIME-DOMAIN FEATURES**			
Short-term variability (STV)	$STV_{j}=\frac{\overset{24}{\underset{i\;=\;2}{\sum }}|T_{j}\left(i\right)-T_{j}\left(i-1\right)|}{23}$, where *T*_*j*_(*i*) is the *i* − *th* sample of the *j* − *th* minute of the interbeat sequence	Variability within 1-min interval due to acceleration and decelarations	Lunghi et al., 2005; Fanelli et al., 2013; Signorini et al., 2014
Interval Index (II)	$II_{j}=\frac{std\left\{\left|dT_{2}|,...,|dT_{24}\right|\right\}}{STV_{j}}$, where *std* is the standard deviation of the sequence composed of the absolute difference of successive samples *T*_*j*_(*i*) − *T*_*j*_(*i* − 1) (|*dT*_*i*_|) of the *j* − *th* minute of the interbeat sequence	Fluctuation between variability of successive beat intervals and STV	Lunghi et al., 2005; Fanelli et al., 2013; Signorini et al., 2014
Long-term variability (LTV)	*LTV*_*j*_ = *max*[*T*_*j*_] − *min*[*T*_*j*_], where *T*_*j*_ is the *j* − *th* minute of the interbeat sequence	Range of the interbeat sequence due to accelaration and decelarations (slow oscillations)	Lunghi et al., 2005; Fanelli et al., 2013
Long-term irregularity (LTI)	LTI is defined as the interquartile range of the following distribution: $m_{k}=\sqrt{\overset{72}{\underset{i\;=\;2}{\sum }}T_{k}^{2}\left(i\right)+T_{k}^{2}\left(i-1\right)},$ where *T*_*k*_(*i*) is the *i* − *th* value of the *k* − *th* 3-min segment of the interbeat sequence	Variability over 3 min intervals (Slow oscillations over longer time scales)	Signorini et al., 2014
STV/LTV	Ratio between STV and LTV	Fluctuation between short and long variation of the interbeat sequence	Reddy et al., 2009
Basal fetal heart rate	Mode of the FHR trace after discarding accelerations and decelerations	Maturity of ANS; as pregnancy advances, the parasympathetic system matures and the FHR is reduced	Signorini et al., 2005
Number of accelerations per minute	Number of accelerations over minutes of the FHR trace	Increment of accelerations due to maturity of vagal functions	Signorini et al., 2005
Acceleration average capacity (AAC)	Calculated following Huhn et al. (2011) with parameters *M* and *L* set to one (parameters were optimized for FHR derived from 1D-DUS signals in Stroux et al., 2016, 2017)	Occurrence or absence of the appearance of accelerations	Fanelli et al., 2013; Signorini et al., 2014
Deceleration average capacity (DAC)	Calculated following Huhn et al. (2011) with parameters *M* and *L* set to one (parameters were optimized for FHR derived from 1D-DUS signals in Stroux et al., 2016, 2017)	Occurrence or absence of the appearance of decelerations	Fanelli et al., 2013; Signorini et al., 2014
Mean (mIs)	Mean of the interbeat-sequence	Maturity of the parasympathetic system	
Standard deviation (stdIs)	Standard deviation of the interbeat-sequence	Dispersion of the interbeat sequence. Variability among interbeat sequence increased as pregnancy progress due to accelerations and decelerations controlled by vagal function	Van Leeuwen et al., 1999; Lange et al., 2005
Variance (varIS)	Variance of the interbeat-sequence	Variability among interbeat sequence	Van Leeuwen et al., 1999; Lange et al., 2005
Root mean square of successive differences (rmssdIS)	Root mean square of successive differences of the interbeat sequence	Variability between successive beat intervals due to accelerations and decelerations controlled by vagal function	Van Leeuwen et al., 1999; Lange et al., 2005
rmssdIS/stdIs	Ration between rmssdIS and stdIs	Fluctuation between variability of successive beat intervals and variability of all the beat intervals	Lange et al., 2005; Tetschke et al., 2016
Skewness (skewnessIS)	Skewness of the interbeat-sequence	Asymmetry on the FHR trace due to accelerations and decelerations controlled by vagal function	Tetschke et al., 2016; Marzbanrad et al., 2017
Kurtosis (kurtosisIS)	Kurtosis of the interbeat-sequence	Occurrence or absence of outliers on the FHR trace due to accelerations and decelerations controlled by vagal function	Tetschke et al., 2016; Marzbanrad et al., 2017
PNN5	The fraction of consecutive beats that differ by more than 5 ms	Formation of accelerations and decelerations controlled by vagal function	Tetschke et al., 2016; Hoyer et al., 2017
**NON-LINEAR AND COMPLEXITY FEATURES**			
Approximate entropy (ApEn)	Calculated with the cardiovascular toolbox (Vest et al., 2018), setting the *m* and *r* parameters in 2 and 0.1 of the standard deviation of the input signal	Regularity and complexity on the interbeat sequence. Complexity increases as gestation progress due to maturation of the parasympathetic system	Van Leeuwen et al., 1999
Fractal dimension	Calculated using the Higuchi's algorithm (Higuchi, 1988), setting the interval parameters as 5	Occurrence of recursive patterns on the interbeat sequence	Kikuchi et al., 2005
Lyapunov exponent	Calculated following steps presented in Rosenstein et al. (1993) setting embedding dimension and lag parameters as 1 and 2	Dynamics properties (contraction or expansion) of the interbeat sequence due to vagal function	Van Leeuwen et al., 1999
Tone	Average of the percentile difference of successive beat intervals	Measurement of the sympatho-vagal balance	Khandoker et al., 2015
Entropy	Calculated by using the Shannon formula (Shannon, 1948) on the percentile difference of successive beat intervals distribution	Complexity of sympatho-vagal balance	Khandoker et al., 2015
Generalized mutual information (GMI)	Calculated following steps in Hoyer et al. (2012), setting dimension parameter at 3 and delay parameter at 1	Complexity of sympatho-vagal balance.	Hoyer et al., 2012, 2017
**FREQUENCY-DOMAIN FEATURES**			
Low frequency (LF)	Power spectral density of the FHR time series on the band 0.03–0.15 Hz	Associated with the sympathetic control and vasomotor activity	Signorini et al., 2003, 2014; Van Leeuwen et al., 2003
Medium frequency (MF)	Power spectral density of the FHR time series on the band 0.15–0.50 Hz	Measurement of the fetal activity and mechanical movement induced by maternal breathing	Signorini et al., 2003, 2014; Van Leeuwen et al., 2003
High frequency (HF)	Power spectral density of the FHR time series on the band 0.5–1 Hz	Associated with respiration controlled by vagal activity	Signorini et al., 2003, 2014; Van Leeuwen et al., 2003
LF/(MF + HF)	Ratio between LF and the summation of MF and HF	Fluctuation between physiological control components and fetus activity level	Signorini et al., 2003, 2014; Van Leeuwen et al., 2003

*The third column provides a physiological interpretation of the feature, and the fourth column displays relevant previous works in which the metrics have used for fetal maturity assessment*.

#### 3.4.2. Features Generated From Maternal Blood Pressure Readings

The maternal systolic blood pressure (SBP), diastolic blood pressure (DBP), and the maternal heart rate (MHR) measurements from the blood pressure device were used as features. Since the SBP, DBP, and MHR were taken for both patient's arms, these values were averaged. The averaged SBP, DBP, MHR were used to generate seven additional hemodynamics formulas, which have been reported to vary throughout pregnancy (Steer et al., 2004; Salas et al., 2006; Kac et al., 2015; Rebelo et al., 2015). Table 4 listed all the features generated from maternal blood pressure readings.

**Table 4:** Detail of maternal hemodynamic formulae calculated using the SBP, DBP and MHR taken with the self-inflating blood pressure device.

**Metric name**	**Formula**	**References**
Pulse pressure (PP)	*SBP* − *DBP*	Stouffer, 2008
Mean arterial pressure (MAP)	(*SBP* + *DBP* × 2)/3	Stouffer, 2008
Cardiac output (CO)	*MHR* × *PP* × 0.002	Hill et al., 2011
Rate pressure product (RPP)	*MHR* × *SBP*	Robinson, 1967
Shock index (SI)	*MHR*/*SBP*	Singh et al., 2014
Modified Shock Index (MSI)	*MHR*/*MAP*	Singh et al., 2014
Stroke volume (SV)	*CO*/*MHR*	Stouffer, 2008

### 3.5. Assessing Potential of Extracted Features

To evaluate the potential of fetal cardiac and maternal blood pressure features to describe the fetal development throughout pregnancy, the Pearson correlation coefficient, and its *p*-value, was calculated for each feature and the gestational age. This rationale was based on previous research articles that have reported linear relationships between cardiac physiological parameters and fetal maturity (Frasch et al., 2007; Hoyer et al., 2019; Signorini et al., 2020). Thus, the correlation coefficients help to check that features used in this work are relevant for GA estimation.

### 3.6. Estimation of Gestational Age

All of the features were extracted for both the 129 normal birth weight and 24 LBW newborns at each stage of pregnancy for which data was available. The GA estimation model was training only with visits of newborns with normal birth weights because previous research has reported that LBW fetuses have discrepancies in their GA estimations from fHRV (Marzbanrad et al., 2017). However, the features derived from the recordings of the LBW newborns were used later to test the model's ability to estimate GA.

The 129 normal weight patients were split into training and test sets. The number of patients for the test set was selected to be proportional to the LBW newborn set. A Wilcoxon rank-sum hypothesis test (two-sided; α= 0.05) was applied in order to test whether there were statistically significant differences between the training and test sets for all the values of the 37 features (if a statistically significant difference was found, the subjects were randomized again).

The training set comprised 104 newborns, from which 95 had one visit, eight had two visits, and one had three visits, giving a total of 114 visits. For the test set there were a total of 25 normal birth weight newborns, for which 22 had one visit and three had two visits, giving a total of 28 visits. Table 5 shows the number of visits for each GA for the training and test sets.

**Table 5:** Number of visits per gestational age (GA) for the 104 normal birth weight training set, the 25 normal birth weight test set, and the 24 low birth weight test set.

**Gestational**	**Training set**	**Test set**	**Test set**
**age (months)**	**Normal birth**	**Normal birth**	**Low birth**
	**weight**	**weight**	**weight**
6	6	3	3
7	28	10	7
8	32	5	5
9	48	10	10
Total	114	28	25

#### 3.6.1. Training/Validation Methodology

The training and validation procedure was performed using a 5-fold cross validation with 50 trials (repetitions). At each trial, the patients were randomly assigned to different folds, ensuring that visit features corresponding to the same patient were in the same folder. Thus, at each iteration, ~20% of the training data was used for validation (held-out fold). By using 50 trials the variability of the models for estimating the GA could be measured, and confidence intervals could be estimated.

Since folds were class unbalanced (i.e., different number of visit for each gestational age), at each iteration of the 5-fold cross-validation, the number of visits per GA was balanced on the training folds before constructing a model. To that end, we used the Adaptive Synthetic Sampling (ADASYN) method, which has been reported to overcome the class imbalance problem in support vector machine models (Batuwita and Paladey, 2013). This method generates synthetic data for the minority classes by taking the Euclidean distance between two data points and then adding the difference scaled by a factor, between 0 and 1, to one of the minority data points. In this study, the ADASYN was implemented as described in He et al. (2008).

Before training a model, the balanced training set and the held-out fold set were standardized by subtracting the mean of the respective feature vector and dividing it by its standard deviation computed in the training data only. This standardization method was selected as it has shown to be suitable for feature scaling in machine learning methods (Tax and Duin, 2000).

The 50-trial 5-fold cross-validation was assessed using three different regression approaches: Elastic Net, Support Vector Regression (SVR), and Gradient Boosting Tree (GBT). At each iteration of the cross-validation, the training folds were used to select the most relevant features for the SVR and GBT models. For the SVR, features were selected using the maximum relevance and minimum redundancy (mRMR) algorithm (Peng et al., 2005), which ranks the most relevant features based on mutual information gain. For the GBT model, the features were selected by training a GBT with 100 trees and learning rate of 1 on the training folds, and then identifying the most relevant features by summing the feature weights over all the weak learners.

To optimize each model's hyperparameters, a nested cross-validation using a grid search on the training folds was used for each model. The grid search for the three models was defined as:

- Elastic Net. The linear penalty term, λ was defined as {0.1, 0.2, ..., 0.8, 0.9}. The quadratic penalty term was given by $\frac{1-\lambda }{2}$. For each λ value, a set of 100 values of regularization parameters were tested. The regularization parameter set was generated by first finding the largest value, θ, that gave a non-null model (i.e., intercept ≠ 0), and then the remaining 99 values were defined by decreasing θ by 10^−5^, so that the ratio of the smallest to the largest value of the set was 10^−4^.
- SVR. The grid search for the soft margin (*C*) and the margin of tolerance (ϵ) were defined as: *C* ∈ {2^−3^, 2^−1^, ..., 2^8^} and ϵ ∈ {2^−10^, 2^−9^, ..., 2^−5^}. The Gaussian radial basis function parameter (γ) was analytically estimated as reported in Caputo et al. (2002). Namely, γ was derived by calculating the distribution of ||*x* − *x*′||^2^ between a subset containing 70% of the training set, and then taking the inverse of the median of this distribution.
- GBT. The grid search for the learning rate was defined as {0.1, 0.25, 0.5, 1} and the number of trees was defined as {100, 150, 200, ..., 500, 550}. The number of maximum splits (tree height) was defined as {1, 2, ..., *log*_2_(*S* − 1)}; where *S* is the total number of visits of the training folds.

#### 3.6.2. Analyzing the Training/Validation Output

The 50 trial 5-fold cross-validation resulted in 50 median absolute error (MAE) vectors, and 250 selected feature vectors. From the 50 MAE vectors, the median, interquantile range, and the lower and upper 95% confidence interval for the median were determined. The median MAEs were compared to select which regression model (Elastict Net, SVR, or GBT) to use in the test stage.

From the 250 selected feature vectors, the top twenty most relevant features were identified. To that end, the features were ranked at each feature vectors, and the mean rank was determined by averaging the ranking of each feature over the 250 feature vectors. This simple aggregation technique was used as it has shown to be effective to combine different features sets in the medical application field (Saeys et al., 2007; Wu et al., 2018).

Using the top ten, top fifteen, and top twenty, the same validation/training procedure was repeated to identify the best performing feature set for estimating GA.

#### 3.6.3. Testing Methodology

The model selected with the best performing feature set was then used to train a final model on the training set of normal birth weight patients. Before training the final model, the training data were balanced using ADASYN (He et al., 2008), and the parameters were optimized using grid search as explained in subsection 3.6.1. The final model was then used to estimate the GA for both the 25 held-out normal birth weight and the 24 LBW patients.

Since the final model depends on the nature of the synthetic data added to the training dataset, the testing procedure was performed 100 times to evaluate the variability of the model's performance. The median, interquantile range, and the lower and upper 95% confidence interval for the median were determined for the two test groups. To determine if there was any difference in GA estimation distribution of errors between the normal and LBW newborns, a two-sided Wilcox rank-sum test hypotheses test was evaluated on the data.

### 3.7. Detecting Possible Cases of IUGR

Since we are assuming that IUGR cases are those with LBW, the estimated GA for the test set were compared against birth weight. To that end, the GA error estimation was defined as the difference between the GA based on the LMP and the median GA estimation over the 100 repetition. Then, a robust least square was fitted using the birth weight as independent variable and the GA error estimation as the response variable.

Finally, the area under the receiver operating characteristic (AUROC) was calculated to discriminate between LBW and NBW newborns. The AUROC was calculated using the difference between LMP-based GA and the estimated GA. As we hypothesized that LBW newborns are likely to result in underestimations (LMP-based GA > estimated GA), a newborn was considered LBW if the difference between the LMP and SVR estimates of the GA was greater than the thresholds used in the AUROC analysis.

## 4. Results

### 4.1. Correlation Between Extracted Feature and Gestational Age

Table 6 shows the Pearson correlation between the features and the gestational age. Nine out of the 37 extracted features showed a statistically significant correlation with gestational age. For the normal birth weight, 38% of the features showed evidence of a significant correlation with gestational age, whereas, for the low birth newborns only two features were correlated. The correlation of fHRV indexes indicated a variation of such metrics during pregnancy, thus being relevant to assess fetal maturation for this project.

**Table 6:** Pearson correlation between the features and the gestational age in months.

**Feature**	**Overall**		**Normal birth weight**		**Low birth weight**	
	**Correlation**	***p*-value**	**Correlation**	***p*-value**	**Correlation**	***p*-value**
STV	−0.03	0.67	−0.08	0.35	0.16	0.45
LTV	0.06	0.45	0.04	0.67	0.14	0.52
AAC	0.11	0.17	0.13	0.11	−0.06	0.78
BHR^§^	−0.03	0.66	0.03	0.70	−0.42	0.04
DBP*^‡^	0.32	<0.01	0.32	<0.01	0.35	0.09
SBP*^‡^	0.30	<0.01	0.32	<0.01	0.16	0.44
MHR^‡^	0.09	0.24	0.16	0.05	−0.29	0.16
DAC	−0.09	0.24	−0.14	0.11	0.17	0.41
#Accelarations/minute*^‡^	0.19	0.01	0.19	0.02	0.20	0.34
II	0.05	0.53	0.07	0.44	−0.06	0.79
LTI*^‡^	−0.24	<0.01	−0.26	<0.01	−0.07	0.73
ApEn	−0.13	0.09	−0.12	0.17	−0.24	0.26
LF	−0.08	0.33	−0.10	0.23	0.08	0.71
MF^‡^	0.14	0.07	0.20	0.02	−0.22	0.29
HF	0.06	0.46	0.08	0.33	−0.09	0.68
LF/(MF + HF)	−0.08	0.31	−0.11	0.19	0.10	0.64
varIS	0.12	0.13	0.11	0.19	0.20	0.33
Fractal Dimension	−0.04	0.61	−0.11	0.19	0.28	0.18
mIS ^§^	0.02	0.79	−0.04	0.61	0.40	0.05
stdIS*^‡^	0.18	0.02	0.18	0.03	0.20	0.34
rmssdIs	0.02	0.84	−0.02	0.80	0.21	0.32
Lyapuno exponent^‡^	0.14	0.07	0.17	0.04	−0.08	0.71
skewnessIs	−0.10	0.20	−0.09	0.28	−0.35	0.09
kurtosisIs	−0.07	0.35	−0.10	0.26	0.11	0.61
GMI	0.09	0.26	0.13	0.14	−0.12	0.58
Tone^‡^	−0.12	0.14	−0.16	0.05	0.19	0.37
Entropy	0.06	0.42	0.04	0.63	0.15	0.48
stdIS/rmssdIs*^‡^	0.20	0.01	0.24	<0.01	0.01	0.96
PNN5	0.07	0.37	0.03	0.69	0.24	0.25
STV/LTV*^‡^	−0.21	0.01	−0.24	<0.01	−0.04	0.85
PP	0.06	0.48	0.08	0.32	−0.11	0.60
MAP*^‡^	0.35	<0.01	0.35	<0.01	0.31	0.13
CO^‡^	0.12	0.11	0.19	0.02	−0.36	0.08
RPP*^‡^	0.22	<0.01	0.30	<0.01	−0.25	0.22
SI	−0.04	0.61	0.01	0.90	−0.30	0.14
MSI	−0.06	0.47	0.00	0.98	−0.35	0.09
SV	0.06	0.48	0.08	0.32	−0.11	0.60

A ^*^, ^‡^, and ^§^ indicate if the correlation coefficient is statistically significant (p-value < 0.05) for overall newborns, normal birth weight newborns, and low birth weight newborns, respectively.

### 4.2. Training/Validation Performance

Table 7 shows the mean absolute error (MAE) for the 50 trial 5-fold cross-validation. For all the three regression models, the MAE of the seventh and eighth gestational months were lower than those of the extreme months evaluated. The regression model with the lowest the overall median MAE over the 50 trials was the SVR with a value of 0.8 months. Furthermore, the SVR and Elastic Net were the models with the lowest interquartile range for the MAE over the 50 trials, thus indicating a lower variance of these to models in comparison to the GBT.

**Table 7:** Mean absolute errors (MAE) of the 50 trial 5-fold cross validation for the Elastic Net, SVR, and Gradient boosting tree.

**Model**	**Metric**	**Gestational age (months)**				
		**6**	**7**	**8**	**9**	**All**
Elastic Net	Median	1.54	0.51	0.50	1.40	0.93
	IQR	0.10	0.04	0.07	0.14	0.08
	LCI	1.52	0.50	0.49	1.36	0.91
	UCI	1.58	0.52	0.52	1.42	0.96
SVR	Median	1.57	0.51	0.43	1.15	0.80
	IQR	0.17	0.09	0.10	0.11	0.06
	LCI	1.54	0.48	0.40	1.11	0.79
	UCI	1.65	0.54	0.47	1.17	0.83
Gradient boosting tree	Median	1.77	0.85	0.65	0.81	0.86
	IQR	0.42	0.20	0.14	0.18	0.08
	LCI	1.66	0.82	0.61	0.77	0.83
	UCI	1.93	0.89	0.70	0.89	0.88

*For each model, the median, interquantile range, and the 95% confidence interval for the median of the MAE are provided*.

### 4.3. Ranking the Features

Table 8 shows the top twenty features for estimating GA based on the average ranking of the 250 feature vectors for the SVR. Seven out of the ten top features were derived from the 1D-Doppler ultrasound, from which fHRV linear indexes were among the most selected. Maternal blood pressure based features were also included in the top features, with MAP as the most selected feature of that group.

**Table 8:** Feature ranking obtained after averaging the individual 250 feature ranking resulted in the 50 trial 5-fold cross-validation.

**Ranking**	**Feature**	**Type**
Top 10	Tone	Non-linear and Complexity
	#acc/min	Linear
	MAP	Hemodynamic formula
	ApEn	Non-linear and Complexity
	KurtosisIS	Linear
	CO	Hemodynamic formula
	STV/LTV	Linear
	SkewnessIS	Linear
	PP	Hemodynamic formula
	LTI	Linear
Top 15	stdIS/rmssdIS	Linear
	DBP	Hemodynamic formula
	RPP	Hemodynamic formula
	varIS	Linear
	stdIS	Linear
Top 20	GMI	Non-linear and Complexity
	II	Linear
	DAC	Linear
	MF	Spectral
	basal FHR	Linear

Table 9 presents the results obtained by repeating the training/validation procedure using the top ten, top fifteen, and top twenty features. The top fifteen feature set achieved the lowest median MAE over the 50 trials with a value of 0.76 months (95% CI = 0.75–0.78 months).

**Table 9:** Mean absolute errors (MAE) of the 50 trial 5-fold cross validation for the SVR using the top10, top15, and top20 features.

**Model**	**Metric**	**Gestational age (months)**				
		**6**	**7**	**8**	**9**	**All**
SVR	Median	1.62	0.48	0.43	1.18	0.82
(top 10 features)	IQR	0.18	0.10	0.09	0.12	0.07
	LCI	1.57	0.45	0.40	1.13	0.80
	UCI	1.67	0.50	0.45	1.21	0.83
SVR	Median	1.51	0.47	0.44	1.04	0.76
(top 15 features)	IQR	0.17	0.08	0.07	0.15	0.07
	LCI	1.44	0.45	0.42	1.01	0.75
	UCI	1.54	0.50	0.46	1.08	0.78
SVR	Median	1.56	0.45	0.43	1.16	0.81
(top 20 features)	IQR	0.15	0.10	0.08	0.09	0.05
	LCI	1.52	0.42	0.40	1.14	0.79
	UCI	1.60	0.47	0.44	1.20	0.81

*For each model, the median, interquantile range, and the 95% confidence interval for the median of the MAE are provided*.

### 4.4. Testing Performance

Table 10 shows the performance of the 100 repetitions of the SVR with the top fifteen tested on the held-out 25 normal birth weight newborns and the 24 LBW newborns. The median MAE for each gestational month and the overall was statistically significantly higher for the LBW newborns (two-sided Wilcox rank-sum test; α = 0.05). The difference between the median MAE for the two groups was increasing throughout the GA, resulting in a difference of 0.29 month for the overall estimation.

**Table 10:** Mean absolute errors (MAE) of the 100 trials on the test (held-out) normal birth weight and LBW newborns.

**Newborn type**	**Metric**	**Gestational age (months)**				
		**6^†^**	**7^†^**	**8^†^**	**9^†^**	**All^†^**
Normal birth weight	Median	1.06	0.53	0.33	0.99	0.72
	IQR	0.25	0.05	0.07	0.07	0.05
	LCI	1.03	0.52	0.32	0.98	0.71
	UCI	1.08	0.54	0.35	1.00	0.72
Low birth weight	Median	1.26	0.73	0.68	1.32	1.01
	IQR	0.11	0.05	0.09	0.06	0.03
	LCI	1.24	0.72	0.67	1.30	1.01
	UCI	1.29	0.74	0.69	1.33	1.02

*For each type of newborns, the median, interquantile range, and the 95% confidence interval for the median of the MAE are provided. A †indicates a significant difference between the median GA estimations of the normal and LBW newborns for the 100 repetitions (two-sided Wilcox rank-sum test; α = 0.05)*.

**Figure 3:**
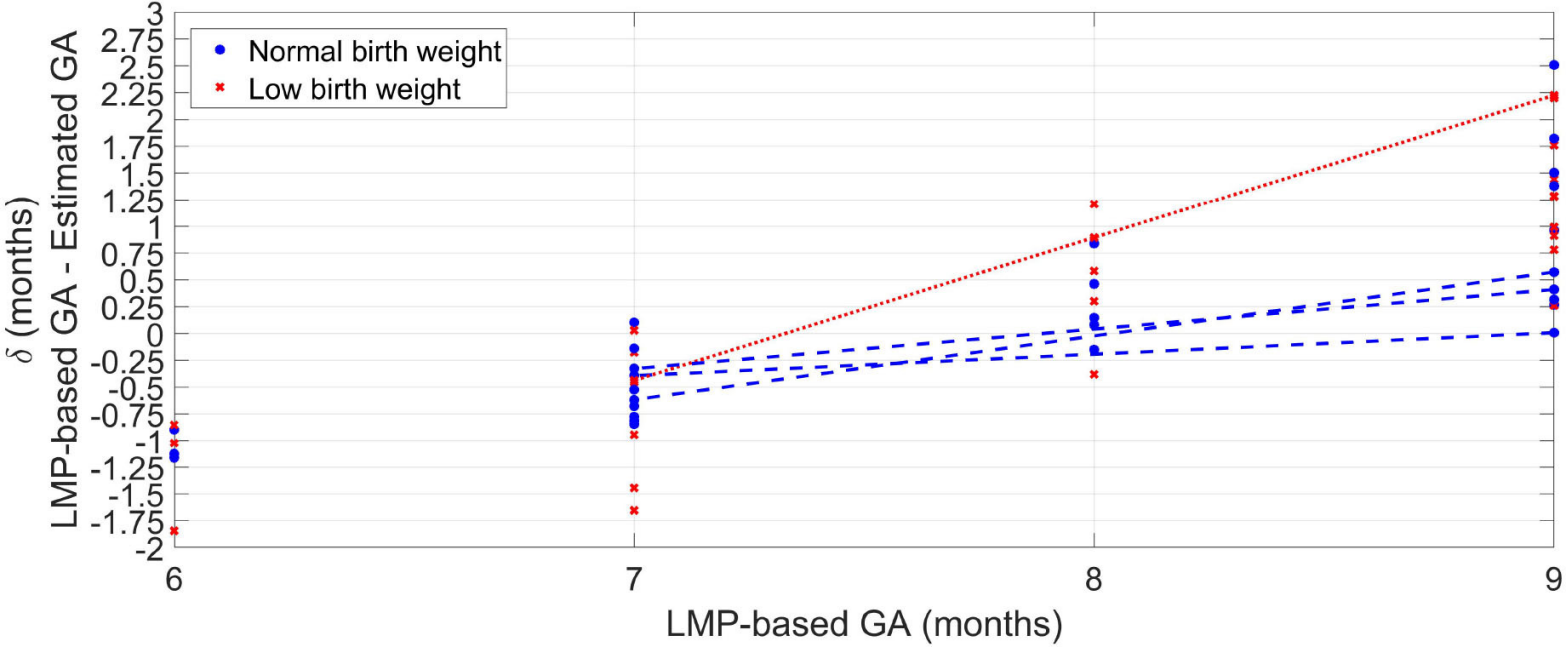
Median error of the 100 repetitions against GA provided by the LMP method for the normal weight newborns (blue circles) and the LBW newborns (red crosses). For newborns with more than one visit, a line connects the median error along with the GA.

Figure 3 shows the difference (δ) between GA based on the LMP and the median estimated GA over the 100 repetitions for each visit. The LBW newborns' (red crosses) GAs were generally overestimated (LMP-GA < estimated GA) for the eighth and ninth gestational months compared to the normal birth weight newborns (blue circles). For the eighth and ninth gestational months, on the other hand, the LBW newborns were generally underestimated (LMP-GA > estimated GA) compared to the normal birth weight newborns.

For newborns with more than one visit, Figure 3 shows a line connecting the median error across GA. For both normal and LBW newborns, the GA estimates trended from overestimation to underestimations as GA increased. However, the discrepancy was higher for the LBW newborn with a difference of around 2.5 months between the seventh and the ninth gestational month. In contrast, the maximum difference for normal birth weight newborns was ~0.75 month.

**Figure 4:**
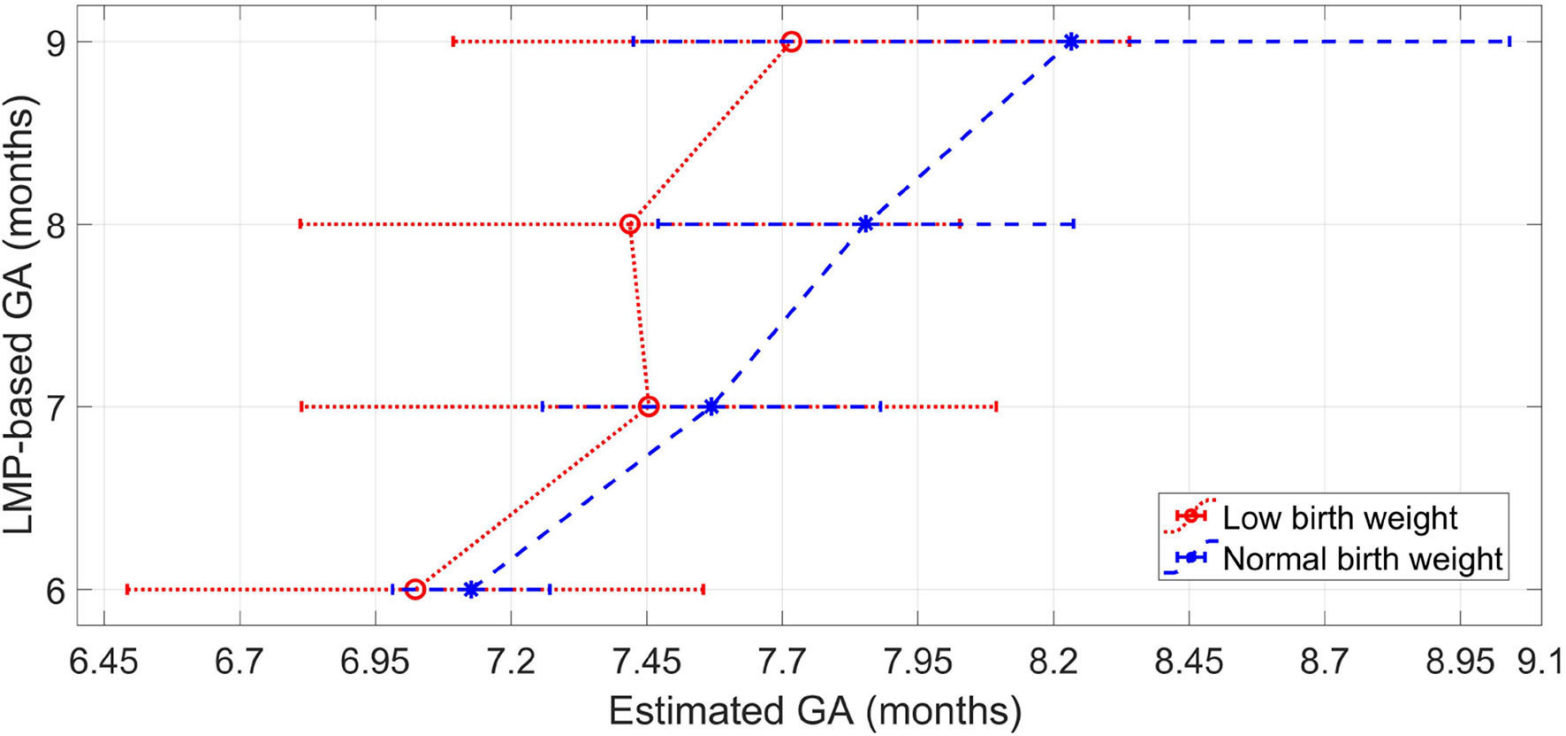
Median and interquartile range of the estimated GA for each label of the LMP method for the normal weight newborns and the LBW newborns.

Figure 4 shows the median of the estimated GA for each label of the LMP method. For all the gestational months, the LBW group resulted in a lower median GA estimations than the normal birth weight group. The median difference between normal and LBW newborns is greater for the last 2 months of pregnancy, thus indicating a higher inconsistency in the features for GA estimation between the type of newborns from the eighth month of gestation onward.

### 4.5. GA Estimation Errors as a Function of Birth Weight

**Figure 5:**
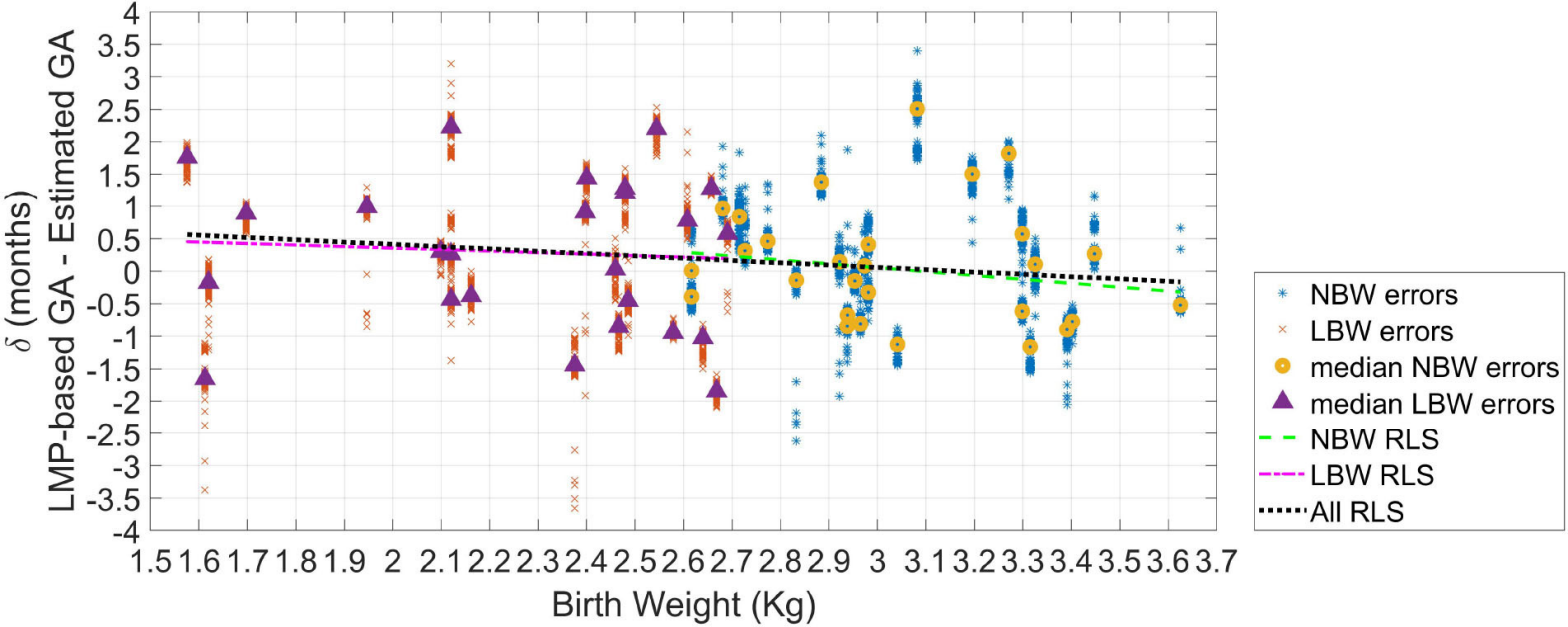
Error in GA from 100 repetitions (δ) as the difference between GA based on the LMP and the estimated GA. The median error of the 100 repetitions for each recording (or visit) is displayed (triangles for LBW newborns; and circles for normal birth weight newborns). Robust least square fits are also shown.

Figure 5 shows the GA estimation errors over estimated birth weight for the tested newborns. Robust least-square fits were performed for each type of newborn, as well as for all the newborns as a whole. All the fits provided negative slopes and negative Pearson correlation values (ρ). The inverse relationship between GA estimation error and birth weight indicates that there are more underestimations for newborns with LBW. In fact, for the LBW newborns, fifteen visits achieved underestimations, whereas ten visits obtained overestimations. For the normal weight newborns, the fitted line was δ_*NBW*_ = 1.85−0.60*w*_*NBW*_(ρ = −0.15, *P*-*value* = 0.45). For the low weight newborns, the fitted line was δ_*LBW*_ = 0.82−0.23*w*_*LBW*_(ρ = −0.06, *P*-*value* = 0.76). For all the newborns, the fitted line was δ = 1.12−0.35*w*(ρ = −0.13, *P*-*value* = 0.37).

The AUROC using the estimation difference between NBW and LBW resulted in 0.55. However, as in Figure 5 is observable than there was a more remarkable difference between newborns with weight lower than 2.3 kg, and those with a weight >3.1 kg, the ROC analysis was repeated for these two groups. This comparison yielded in an AUROC of 0.63, thus suggesting acceptable discrimination for these two groups.

## 5. Discussion

### 5.1. Interpretations of Findings

The results presented in this work indicate that it is possible to provide a proxy for screening fetal growth retardation in a resource-constrained setting by using the difference between GA estimated by LMP and the GA estimated from features extracted from an inexpensive Doppler transducer and a blood pressure device.

This proxy fetal assessment relies on the GA estimation approach introduced in this work, which using a pregnancy conversion factor of $\frac{40weeks}{9months}$ resulted in a median MAE of 3.2 and 4.5 weeks for the normal and LBW newborns, respectively. Interestingly, these MAE values are comparable to those presented in Marzbanrad et al. (2016) and Marzbanrad et al. (2017) of 2.7–5.1 weeks obtained using a step-wise regression using 1D-DUS and fECG signals recorded by medical professionals in a high-resource/high-income country. Moreover, unlike Tetschke et al. (2016), our work did not require high-resolution input signals, making the implementation of the approach described here feasible in LMICs. Notably, our GA estimations were lower than the 6 weeks error associated with symphysis-fundal height, which is the common dating method used in LMICs to detect IUGR (Griffiths et al., 2008).

The higher GA estimation errors for the LBW newborns indicate that this type of patient has different patterns in the 1D-DUS and maternal blood pressure features than normal birth weight newborns of the same GA. We note that this difference is related to the birth weight as the AUROC between very low birth weight (<2.3 kg) and normal birth weight indicated acceptable discrimination. Therefore, assuming that LBW is a consequence of IUGR (Lee et al., 2013), a potential sign of IUGR can be detected when the estimated GA is lower than the GA calculated from the LMP. This provides evidence to indicate that our method is an alternative and low-cost fetal growth assessment approach for identifying cases that need to be referred for further medical assessment in LMICs, in which symphysis-fundal height measurement is not sufficiently accurate (World Health Organization, 2016), and ultrasound imaging is not available (World Health Organization, 2014).

The longitudinal changes in the difference between GA estimations of low and normal birth weight newborns across gestation suggests that IUGR is progressive and is more evident for the eighth and ninth gestational months, as is shown in Figure 4 for patient with more than one visit. Therefore, our proxy method may be more effective in detecting fetal growth abnormalities during the last 2 months of gestation, thus helping to identify fetuses that need assistance during delivery to reduce adverse perinatal outcomes.

Another interesting finding was the selected features for estimating GA. The top fifteen features (Table 8) were consistent with features previous work for assessing gestational development (Van Leeuwen et al., 1999, 2003; Signorini et al., 2003; Wakai, 2004; Lange et al., 2005; Khandoker et al., 2009). Specifically, linear, non-linear and complexity features, such as tone, the number of accelerations per minute, approximate entropy, and statistical moments of the interbeat sequence, were the features which provided the SVR with the highest performance boost. The feature selection algorithm also demonstrated the potential of blood-pressure-derived features. This selection was relevant as little research has used this type of features for assessing fetal maturity. Finally, the STV/LTV ratio, which previously has shown to be relevant for detecting IUGR cases (Stroux et al., 2017), was also relevant for GA estimation.

### 5.2. Study Limitations

It should be noted that in this work possible cases of IUGR were defined by newborns birth weight. This assumption could not be validated as patients did not receive an ultrasound imaging exam to detect IUGR based on obstetrician standards. Moreover, LBW also includes small for gestational age and appropriate for gestational age/premature newborns (<37 weeks). Presumably, premature and IUGR newborns behave similarly, and there is overlap between these two groups (Lee et al., 2013). Thus, our proxy method may be affected because LBW newborns do not fully capture all the IUGR population since such definition misses infants born small for gestational age above the 2,500 g cutoff and those who are both preterm and small for gestational age. Nevertheless, using LBW as a surrogate for IUGR was based on the fact that in LMICs around 60% of the LBW is caused by IUGR (Lee et al., 2013).

The method presented here estimates GA using the LMP method as a reference. As LMP is not a completely unbiased method for dating fetuses (Andersen et al., 1981; Dietz et al., 2007), our results may contain a bias. Moreover, since the errors in GA estimation provided by our method were larger than 2 weeks [the estimation error recommended in the literature (MacGregor and Sabbagha, 2008)], our method is not accurate enough to be used as a primary method for dating GA. However, for rural areas in LMICS, in which there is a lack of ultrasound imaging equipment and obstetricians, our method is a proxy for screening fetuses with possible abnormalities (LBW or IUGR) that need to be referred for further medical diagnosis and treatment. Superiority to the symphysis-fundal height measurement indicates that this method should be preferred.

Another limitation of this work was that the GA was recorded by the clinical team in months rather than weeks (Martinez et al., 2017). However, as a month includes a variable number of days, this introduces a quantization/rounding error—fetuses just a few days apart that fall into different months will look similar but will be identified as different. This decreases the accuracy of any model fitted to the data, resulting in larger absolute errors for the sixth and ninth gestational months.

This error can be thought of as a higher intra-class variance. When intra-class variance is high, it is recommended to use a longitudinal approach rather than cross-sectional one (Diggle et al., 2002). However, those models need multiple points per subject, in order to be able to apply mixed models considering the random effects of each individual. In this study we could not apply a longitudinal approach because the majority of subjects contained only one valid visit. Nevertheless, the MAE values obtained for our approach suggests that features and methods used here are promising for estimating GA based on the LMP method, which is a low-cost, feasible method to date pregnancy in LMICs (Neufeld et al., 2006).

Our study also included visits that were between the sixth and ninth months of gestation. To fully assess the capacity of our approach to estimating GA, it should be evaluate on metrics recorded in the first and second trimesters. Such an evaluation would allow for the comparison of our GA estimation in a fetal development period in which genetic and biological variability of fetal size is low, and in which Doppler images methods estimate GA more accurately (Reece et al., 1989).

We note that the analysis in this study applies only to singleton pregnancies. Non-singleton pregnancies were not assessed in this study and growth rates observed in these fetuses may well be divergent, or asymmetric.

Finally, we note that the approach presented here did not consider fetal sex to estimate GA. Although it may influence fHRV metrics used here for GA estimation, we deliberately avoid gender because the aim is to avoid the use of imaging Doppler, and sex determination, which present significant cost and social problems, respectively.

### 5.3. Future Directions

Future research should focus on increasing the temporal resolution of the GA labels (by recording the week of the LMP through community surveys perhaps), and use a more accurate dating method, such as expert-driven ultrasound imaging.

Future research should also evaluate the efficacy of the proxy presented here on confirmed diagnoses of IUGR. This evaluation would allow a full end-to-end assessment of how 1D-DUS and maternal blood pressure can contribute to detection fetal growth abnormalities.

Moreover, future research should ensure the collection of multiple visits during the course of pregnancy (and extending this to earlier gestational periods), so that a longitudinal analysis can be performed that incorporates the individual dynamics into the model.

## 6. Conclusion

This work introduced a proxy to detect possible cases of IUGR for constrained-resource environments in which ultrasound imaging is not available, and current low-cost methods are prone to error. The potential IUGR cases are detected by comparing GA based on the last menstrual period with estimates obtained 1D-DUS and maternal blood pressure recordings collected with inexpensive devices, usable with little training. The method is valuable to endow non-medically trained operators with an objective metric to identify cases that need to be referred to further medical assistance. The assessment system may, therefore, have an immediate impact if coupled with suitable intervention, such as nutritional supplementation. However, a prospective clinical trial is required to show the efficacy of such metrics and intervention.

## Data Availability Statement

The datasets generated for this study will not be made publicly available. The data was collected in Guatemala rural community, including recordings of a vulnerable indigenous population. We need to obtain permission from the ethical committee before making the dataset publicly available.

## Ethics Statement

The studies involving human participants were reviewed and approved by the Institutional Review Boards of Emory University, the Wuqu' Kawoq|Maya Health Alliance, and Agnes Scott College (Ref: IRB00076231—Mobile Health Intervention to Improve Perinatal Continuum of Care in Guatemala) and registered on ClinicalTrials.gov (identifier NCT02348840). The patients/participants provided their written informed consent to participate in this study.

## Author Contributions

GC, RH-C, and PR conceived of the study and acquired the funding. RH-C and PR coordinated the data collection process in the Guatemalan rural community. CV, FM, and GC contributed to the development of techniques and analysis tools. The data was analyzed by CV, FM, and GC. CV implemented the methods presented here for estimating gestational age. CV, FM, PR, and GC contributed to the writing of the manuscript. All authors reviewed and approved the final manuscript.

## Conflict of Interest

The authors declare that the research was conducted in the absence of any commercial or financial relationships that could be construed as a potential conflict of interest.

## Abbreviations

AAC: acceleration average capacity
ADASYN: adaptive synthetic sampling
ANS: autonomic nervous system
ApEn: approximate entropy
AUROC: area under the receiver operating characteristic
BPM: beat per minute
CO: cardiac output
DAC: deceleration average capacity
DBP: diastolic blood pressure
DUS: Doppler ultrasound signal
FHR: fetal heart rate
fHRV: fetal heart rate variability
GA: gestational age
GBT: gradient boosting tree
GMI: generalized mutual information
HF: high frequency (0.5–1 Hz)
II: interval index
IUGR: intrauterine growth restriction
LBW: low birth weight
LF: low frequency (0.03–0.15 Hz)
LMICs: low- and middle-income countries
LMP: last menstrual period
LTV: long term variability
MAE: mean absolute error
MAP: mean arterial pressure
MF: medium frequency (0.15–0.5 Hz)
mIS: mean of the interbeat sequence
MHR: maternal heart rate
mRMR: minimum redundancy and maximum relevance algorithm
MSI: modified shock index
NBW: normal birth weight
PNN5: percentage of consecutive beats that differ by more than 5 ms
PP: pulse pressure
rmssdIs: root mean square of successive differences
RPP: rate pressure product
SBP: systolic blood pressure
SI: shock index
stdIS: standard deviation of the interbeat sequence
STV: short term variability
SV: stroke volume
SVR: support vector regression
varIS: variance of the interbeat sequence.
